# Bioactive Components and Anticancer Activities of Spray-Dried New Zealand Tamarillo Powder

**DOI:** 10.3390/molecules27092687

**Published:** 2022-04-21

**Authors:** Qian Liu, Nazimah Hamid, Ye Liu, Rothman Kam, Kevin Kantono, Kelvin Wang, Jun Lu

**Affiliations:** 1 Department of Food Science and Microbiology, Faculty of Health and Environment Sciences, Auckland University of Technology, Private Bag 92006, Auckland 1142, New Zealand; qian.liu@aut.ac.nz (Q.L.); ye.liu@aut.ac.nz (Y.L.); rothman.kam@aut.ac.nz (R.K.); kevin.kantono@aut.ac.nz (K.K.); 2 School of Science, Faculty of Health & Environmental Sciences, Auckland University of Technology, Auckland 1142, New Zealand; kelvin.wang@aut.ac.nz (K.W.); jun.lu@aut.ac.nz (J.L.); 3 School of Public Health & Interdisciplinary Studies, Faculty of Health & Environmental Sciences, Auckland University of Technology, Auckland 1142, New Zealand

**Keywords:** tamarillo, spray-drying, polyphenols, anticancer, antioxidant, response surface methodology

## Abstract

Tamarillo fruit contains many phytochemicals that have beneficial therapeutic and nutritional properties. Spray-drying is widely used to preserve fruit puree in powder form. However, to obtain high-quality fruit powder, the optimisation of spray-drying conditions is necessary, as a high drying temperature can damage sensitive bioactive compounds. This study investigated the effects of spray-drying on the microstructure, polyphenolics, total flavonoids, total carotenoids, antioxidant activity, and anticancer capacity of tamarillo powder. Response surface methodology (RSM) was used to optimise the spray-drying process to produce tamarillo powder. The independent variables were inlet drying temperature (120–160 °C), flow rate (1–5 g/mL), and maltodextrin concentration (0–10%). These variables influenced the microstructural attributes, bioactive components, and cytotoxicity of the spray-dried tamarillo powder. The increase in polyphenols and antioxidant activities were favoured under high-temperature spray drying conditions and a low carrier concentration. The optimised spray-drying conditions for producing tamarillo powder with high antioxidant and anticancer activities, high yield, and stable bioactive compounds were found to be at 146.8 °C inlet temperature, and a flow rate of 1.76 g/mL.

## 1. Introduction

Tamarillo is a fruit from a small tree in the flowering plant family *Salicaceae*. It is also known as the tree tomato because its flesh is very similar to that of tomatoes [1]. This subtropical fruit is native to the Andes region, and in New Zealand, it is grown mainly in the North Island. The fruit can be eaten directly or processed into juice and desserts. New Zealand is one of the major producers of tamarillo, and its major export markets include North America, Oceania, and Asia. In New Zealand, tamarillo comes in yellow, red, and fuchsia colours. However, red tamarillo is more popular and common than the other varieties [2].

Tamarillo is a fruit with powerful antioxidant properties. Mutalib et al. [3] reported that the scavenging effect of tamarillo was attributed to its total phenolic content. It was found that the *n*-butanol extract of tamarillo had a strong DPPH free-radical scavenging activity of 0.70 mg/mL. Diep et al. [4] used the CUPRAC and FRAP methods to further determine the antioxidant activities of different species of tamarillo. They reported that the New Zealand purple–red Mulligan tamarillo had the highest antioxidant capacity. The authors also found a strong correlation between total phenolic content and antioxidant activity. Atiqah et al. [5] further confirmed that tamarillo contained a high total phenolic content (7.63 ± 0.37 mg GAE/g), and that there was also a positive correlation between antioxidant activity and total phenolic content.

Tamarillo is a fruit rich in polyphenols that have anticancer properties. In New Zealand tamarillo, a total of 12 polyphenols has been identified [4], with chlorogenic acid being the most abundant polyphenol present. Phenolic antioxidants that reduce oxidative stress can have anticancer activities [6]. The main mechanism of suppressing tumours is by inducing apoptosis that inhibits the promotion and progression of cancer. Chlorogenic acid can stimulate apoptosis and inhibit cell proliferation by inducing mitochondrial-dependent pathway expression [7]. It is capable of eliminating lung cancer stem cells [8,9]. Hydroxycinnamic acid and hydroxybenzoic acid are also present in New Zealand tamarillo fruit [4]. Hydroxycinnamic acid can promote apoptosis of cancer cells by scavenging free-radical activity [10]. It was found that hydroxycinnamic acid inhibited the excessive proliferation of epithelial cells and prevented the development of colon and cervical cancers. In addition, tamarillo extracts have been found to have selective cytotoxicity towards liver hepatocellular carcinoma (HepG2) and non-hormone-dependent breast carcinoma (MDA-MB-231) [11]. These findings suggest that the tamarillo is potentially a good anticancer agent.

In the food industry, spray-drying technology is often used to convert fruit puree or extracts into powder. Spray-drying can extend the shelf life of biologically active substances, protect natural extracts, permit the diffusion and evaporation of volatile compounds to the environment, and improve the stability of the core material by protecting it from irreversible damage caused by oxidation [12]. It is extensively used in food industries to produce fruit powders as it is economical. A review by Shishir and Chen [13] reported that the most significant factors in spray-drying that influenced the quality of fruit and vegetable powders are inlet temperature and carrier agents. The authors concluded that inlet air temperatures of between 120–180 °C, and a maltodextrin concentration of between 7–20% are the most common spray-drying conditions for producing fruit and vegetable powders. They found that although a higher drying inlet temperature can increase yield, Tg, solubility, hygroscopicity, and particle size, it can reduce moisture content, water activity, bulk density, and bioactive compounds. Higher temperatures can result in the powder sticking, which can influence solute activity and antioxidant components. The protection of bioactive compounds can be improved by using carrier agents such as maltodextrin or a combination of maltodextrin and gum Arabic. Turkiewicz et al. [14] found that maltodextrin resulted in powders that had the highest total phenol content compared to powders with inulin and maltodextrin (1:2). Results reported by Jokić et al. [15] further showed that when maltodextrin was used as the carrier material, the retention rate of phytochemicals was about 74%. 

Antioxidant compounds in foods have limited health benefits due to their weaker stability with processing, distribution, and storage conditions [16]. Hence microencapsulation of fruit extracts containing various nutrients and bioactive compounds has become the focus of secondary processing. Processing technologies that can retain phytochemical activities and extend the shelf-life of fruits have the potential to expand the market for fruit products [17]. The effects of freeze-drying and spray-drying on phytochemicals [18], and the inhibitory effects on cancer cells after microencapsulation [19], have also been investigated. Hence, this study was carried out to determine and optimise the spray-drying conditions of tamarillo puree using the response surface methodology (RSM) to produce powders with high antioxidant and anticancer activities.

## 2. Materials and Methods

### 2.1. Chemicals and Reagents

All chemicals and reagents were obtained from Sigma-Aldrich (Auckland, New Zealand). All the chemical standards were purchased from Extrasynthese (Geney, France). Milli-Q water used in this study was produced using a Milli-Q water purification system (Merck Millipore, MA, USA). Human breast cancer cells (MCF-7 and MDA-MB-231), and human melanoma (Malme-3M) cells were obtained from ATCC In Vitro Technologies (Auckland, New Zealand). The cells were cultured in RPMI-1640 medium (no glutamine) containing 10% fetal bovine serum (Malme-3M is 20% fetal bovine serum), 1% L-glutamine (200 mM), and 1% antibiotics (Penicillin–Streptomycin (10,000 U/mL)), in a 75 cm^2^ flask containing 5% CO_2_, and incubated in humidified air at 37 °C. All cells and consumables used in the experiment were purchased from Life Technologies, New Zealand Pty Ltd.

### 2.2. Spray-Drying of Tamarillo Fruits

Tamarillo was harvested 24 weeks into the season and picked at full maturity from Ngapuke Orchards, Matamata, New Zealand. The fruits were thoroughly washed to remove dust and debris from the surface, weighed, and pureed after peeling. The puree was filtered prior to spray-drying. A laboratory-scale Buchi mini spray-dryer B-290 equipped with a Buchi B-296 dehumidifier (Hendrik-Ido-Ambacht, The Netherlands) was used in this study to remove all moisture in the spray-drying air. The spray-dryer unit was coupled with a 0.70 mm spraying nozzle. Maltodextrin was used as a carrier. 

The spray-drying conditions, which included temperature, flow rate, and carrier concentration, were the dependent variables used in this study. The experimental design used was based on RSM, and applied to investigate the influence of temperature (120–160 °C), flow (1–5 g/mL), and carrier (0–10%) on the bioactive components and anticancer activities of the spray-dried tamarillo powder. A total of 15 samples (Table 1) were formulated based on the Box–Behnken design with three replicates. The spray-dried fruit juice powder was collected at the bottom of the cyclone jar. The samples were then transferred to paper foil polyethylene packages of size 15 cm × 20 cm, sealed immediately, and stored in the freezer (−20 °C) until further analysis. A second-order polynomial, shown in the equation below, was assumed to relate the response (Y):

Y = λ_1_X_1_ + λ_2_X_2_ + λ_3_X_3_ (linear) (1)

Y = λ_1_X_1_ + λ_2_X_2_ + λ_3_X_3_ + (λ_1_X_1_)^2^ + (λ_2_X_2_)^2^ + (λ_3_X_3_)^2^ (squared) (2)

Y = λ_1_X_1_ + λ_2_X_2_ + λ_3_X_3_ + λ_1_X_1_λ_2_X_2_ + λ_1_X_1_λ_3_X_3_ + λ_2_X_2_λ_3_X_3_ (2-way interaction) (3)

where Y represents the responses of the experiment, X_1_ is defined as temperature, X_2_ defined as flow rate, X_3_ defined as carrier, and λ is the constant coefficients.

### 2.3. Scanning Electron Microscope

As the spray-dried samples contained some moisture, the powder was freeze-dried prior to SEM analysis. The dried samples were covered with platinum for 100 s at room temperature with a 25-mA discharge current ion sputter (Hitachi E-1045, Tokyo, Japan). The morphological structures of the tamarillo powder were observed using a scanning electron microscop (Hitachi SU-70 field Emission SEM, Tokyo, Japan) at 10.0 kV and magnification at 450×.

### 2.4. Preparation of Tamarillo Extracts

Tamarillo powder (~1 g) was extracted with 4 mL of 50% methanol at room temperature for 1 h and centrifuged at 1500 rpm for 20 min. The remaining residue in the centrifuge tube was re-extracted using 4 mL of 70% acetone at room temperature for 1 h, followed by centrifugation. The supernatant from both extractions was collected and made up to a total volume of 10 mL using deionised distilled water.

### 2.5. Ferric Reducing–Antioxidant Power (FRAP) Assay

The FRAP antioxidant method was carried out according to Rezende et al. [20] with some modifications. For preparation of the FRAP reagent, 10 mL acetic acid buffer (300 mM) and 1 mL TPTZ (10 mM) were freshly prepared, daily, then 1 mL FeCl_3_ (20 mM) in pure distilled water was added and heated to 36 °C before use. 100 µL of the extract was diluted 10× by adding 900 µL distilled water. The FRAP reagent (2 mL) was added, vortexed for 5 s, and kept at room temperature for 4 min. The solution was placed into a cuvette and the absorbance read at 593 nm using a UV/VIS spectrophotometer (Ultrospec 2100 pro UV/visible spectrophotometer, Harvard Bioscience, Holliston, MA, USA). Results were reported in mg Trolox equivalents (TE)/100 g of tamarillo powder using a standard curve prepared using Trolox (0–160 ppm).

### 2.6. Cupric-Reducing Antioxidant Capacity (CUPRAC) Assay

The CUPRAC reducing absorbance capacity assay was performed according to Assefa et al. [21]. An amount of 100 µL of extract was diluted 10× by adding 900 µL distilled water. Then, 1 mL each of 0.01 M CuCl_2_, 1.0 M ammonium acetate, and 7.5 mM neocuproine were added. After incubation at room temperature for 4 min, the absorbance was measured at 450 nm. Antioxidant activity was calculated from a standard curve prepared using Trolox (0–80 ppm) and expressed in mg TE/100 g of powder.

### 2.7. Total Phenolic Content (TPC)

Soluble phenol analysis of the tamarillo powder extract was determined according to the method described by Belwal et al. [22]. The extract (100 µL) was diluted 10 × by adding 900 µL distilled water. Then, 500 µL of Folin–Ciocalteu’s Phenol reagent was added and kept for 5 min. An amount of 1.5 mL of sodium bicarbonate (20% *w*/*v*) was further added to the solution. The mixture was kept in the dark at room temperature for 90 min, and the absorbance measured using a UV/VIS spectrophotometer (Ultrospec 2100 pro UV/visible spectrophotometer, Harvard Bioscience, Holliston, MA, USA) at 765 nm against a blank sample. The total phenolic content was quantified based on a standard curve of gallic acid (0–80 ppm) prepared in methanol. The results were expressed as mg gallic acid equivalents per 100 g dry weight (mg GAE/100 g DW) of tamarillo powder.

### 2.8. Phosphomolybdenum Total Antioxidant Capacity

For the phosphomolybdenum assay, the procedure by Prieto et al. [23] was followed with some modifications. 100 µL of extract was diluted 10 × by adding 900 µL distilled water. Then, 2.8 mL monopotassium phosphate (0.1 M), 6 mL of 1 M sulphuric acid, 0.4 mL of ammonium heptamolybdate (0.1 M), and 0.8 mL of distilled H_2_O were added in a vial and mixed well using a vortex mixer. The vials were placed in an oven at 90 °C for 90 min, and then cooled quickly in an ice bath until the samples turned dark blue or green. The absorbance was measured using a UV/VIS spectrophotometer (Ultrospec 2100 pro UV/visible spectrophotometer, Harvard Bioscience, Holliston, MA, USA) at 700 nm against a blank sample. The total antioxidant capacity was quantified based on a Trolox standard curve (12.5–400 ppm), and the results expressed in mg TE/100 g of powder.

### 2.9. Total Flavonoids

Total flavonoids was determined using the aluminium chloride colorimetry method (Moo-Huchin et al. [24]), and quercetin was used as the standard. 100 µL of extract was diluted 10 × by adding 900 µL distilled water. Then, 4 mL water and 300 µL 5% NaNO_2_ were added, and vortexed for 5 s. The solution was kept at room temperature for 5 min. 10% AlCl_3_ in methanolic solution (300 µL) and 1 M NaOH (2 mL) were added, and vortexed for 5 s. The solution was made up to 10 mL with deionised water. The sample absorbance was then measured at 415 nm relative to the control sample. The content of total flavonoids was calculated and expressed as mg Quercetin equivalents (QEs)/100 g DW, with quercetin used as the standard material. The standard curve of quercetin (0–60 ppm) was prepared.

### 2.10. Total Carotenoids

The total carotenoid content of tamarillo powders was determined spectrophotometrically according to Moo-Huchin et al. [24] with minor modification. A mixed solvent of hexane, acetone, and ethanol in a ratio of 70:15:15 (50 mL) was added to tamarillo powder (0.5 g) containing 0.05% BHT. The mixture was shaken using a vortex mixer for 1 h. Then, 40% KOH in methanol solution (5 mL) was added and incubated at room temperature for 2 h in the dark. The extraction was repeated three times by adding hexane (15 mL) each time. The lower layer was combined and filtered using sodium sulphate powder to remove traces of water. The solutions were measured at 450 nm using a UV/VIS spectrophotometer. β-carotene (0–50 ppm) was used as a standard, and the results were expressed as mg β-carotene/100 g of tamarillo powder.

### 2.11. LC-MS Analysis of Polyphenols

The polyphenolic composition of tamarillo powder was analysed according to Assefa et al [21] with some modifications. Phenolics were qualitatively and quantitatively analysed by using an Agilent 6420 Accurate Mass Quadrupole Time-of-Flight LC/MS, and a model G1978B multimode ionisation source with an Agilent 1260 Series Rapid Resolution LC System (Santa Clara, CA, USA). The separation of polyphenols was performed on a Symmetry C18 column (2.1 × 100 mm, 1.7 µm) with a flow rate of 0.25 mL/min and column temperature of 25 °C. The mobile phase consisted of (A) HPLC grade water + 0.1% formic acid, and (B) acetonitrile + 0.1% formic acid. A linear gradient program was used: 97% A/3% B at 0 min and held for 0.5 min; 85% A/15% B at 0.5–1 min; 75% A/25% B at 1–6 min; 65% A/35% B at 6–8 min; 50% A/50% B at 8–9 min and held for 2 min; 20% A/80% B at 11–12 min and held for 1 min. The total run time was 23 min. The conditions for the MS analyses were: drying gas (N_2_), temperature at 300 °C, drying gas flow at 10 L/min, nebuliser pressure at 40 psi, and the capillary voltage of 4 kV. For the quantification of individual phenolics in tamarillo powder, the standards used for comparison and identification included gallic acid, chlorogenic acid, ferulic acid, caffeic acid, ellagic acid, isorhamnetin, rutin, quercetin, kaempferol rutinoside and kaempferol.

### 2.12. MTT Assay

The MTT method was performed according to Mutalib et al. [3]. Log phase MDA-MB-231, MCF-7, and M-3M cell suspensions (1 × 10^3^ cell/mL) were seeded into a 96-well culture plate. RPMI 1640 medium was used for serial double dilution of tamarillo powder to give concentrations of 5, 2.5, 1.25, 0.625, 0.3125, 0.1563, 0.07813, 0.03906, and 0.01953 mg/mL. With M-3M cells, a 20% fetal bovine serum medium was used for dilution and further incubated at 37 °C for 48 h. Before adding the MTT solution, all the old medium containing tamarillo sample was removed, and 100 μL of a new complete medium was added into each well of the plate. Then, 10 μL of MTT reagent was added to each well, and the plate was incubated for 4 h. The growth media (80 μL) were subsequently removed and replaced with 100 μL of dimethyl sulfoxide (DMSO). The absorbances of the samples were measured at 540 nm.

### 2.13. Statistical Analysis

All experiments were carried out in triplicate and reported as mean ± standard deviation of the mean. A repeated measures Analysis of Variance (RM-ANOVA) was carried out with degrees of freedom adjusted using the Greenhouse–Geisser correction if the sphericity assumption was violated. Fisher’s Least Significant Difference (LSD) post hoc test was also carried out when a response reached significance. All univariate analyses were carried out using Minitab version 19.2020.1 (Minitab Ltd., State College, PA, USA), with an alpha level of 5% (*p* < 0.05). A multivariate approach using Partial Least Squares Regression (PLSR) was carried out to determine the relationship between the polyphenols, antioxidant activities, and anticancer activities of spray-dried tamarillo powder. Variable Importance in Projection (VIP) scores were first calculated to understand the importance of polyphenols and antioxidant activities in relation to anticancer activity. A VIP above 0.8 (90% CI) was considered important, while a VIP above 1 (95% CI) was considered to be most important [25]. A correlation plot further summarised the relationship between antioxidant and anticancer activities. All multivariate analyses were carried out using XLSTAT version 2021.1 (Addinsoft Inc., Paris, France).

## 3. Results and Discussion

### 3.1. Surface Morphology of Tamarillo Powder (SEM)

**Figure 1:**
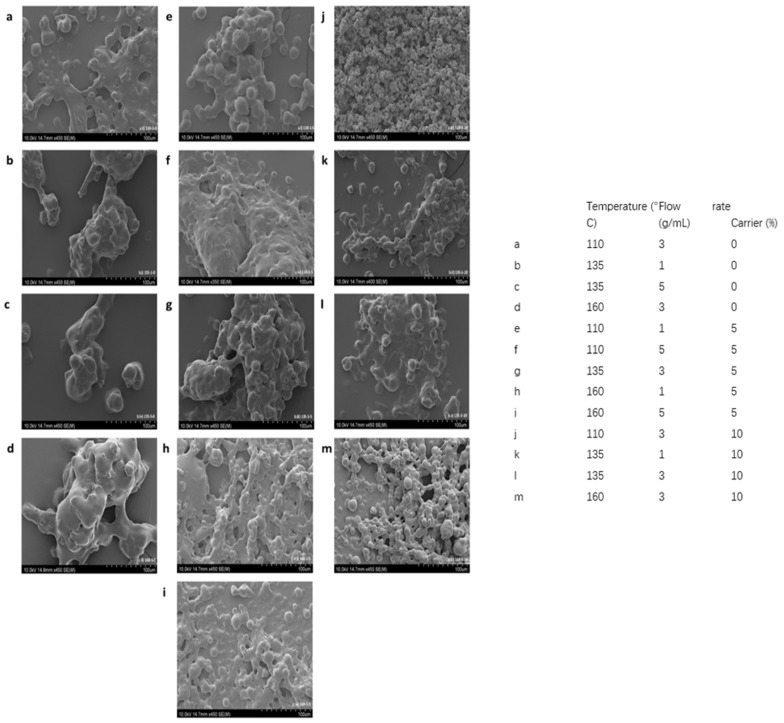
The microstructure of Tamarillo powder under different spray-drying conditions (temperature, flowrate, and carrier concentration). Powder microstructure shown in (**a**–**m**) varied with spray drying conditions summarised in the table.

The morphological structures of tamarillo powder microencapsulated under different spray-drying conditions are shown in Figure 1. Tamarillo powder had a polydisperse spherical structure with a smooth, but not uniform, surface. The inclusion of a carrier during spray-drying can affect the degree of microencapsulation of fruit powders and result in a high degree of globular structure and uniform drying, as well as improved mobility of agglomerated particles [26]. Notably, the degree of microencapsulation had a direct relationship with the carrier concentration in this study. Without the maltodextrin carrier, the tamarillo powder did not form a single globular structure or spherical microparticles when encapsulated (Figure 1a–d). Instead, the particles agglomerated together to form large particles. High-viscosity solid powder can easily adhere to the wall of the main chamber of the spray-dryer to form a solid paste, which reduces the powder yield [27].

At a carrier concentration of 5%, tamarillo powder had more spherical microcapsules with a smooth surface and a moderate level of agglomeration (Figure 1e–i). When the carrier concentration was increased to 10%, the spherical microparticles had a smaller mean particle size and a lower agglomeration level (Figure 1j–m). According to Luis Villacrez et al. [28], maltodextrins are high-molecular-weight wall materials that prevent microcapsules’ surfaces from shrinking, and also reduce the contact of polymer chains and the rigidity of the cladding structure. The effect of temperature at 0% (Figure 1a–d), 5% (Figure 1e–i) and 10% (Figure 1j–m) carrier concentrations was evident. With increasing carrier concentration, the particle morphology improved. Samples at the same temperature but at a higher carrier concentration (10%) resulted in fewer particles adhering to each other, with many separate spherical particle structures. Moreover, the adhesion of particles to each other, as well as the formation of solid bridges between them, was less than in samples with a low carrier concentration or without a carrier. However, spray-drying at a high temperature (160 °C) and at a high carrier concentration of 10% (Figure 1m) resulted in lower production efficiency and powder loss due to sticking. At a high carrier concentration (10%) and a low temperature (110 °C), there was less sticking of particles, which results in better flowability.

### 3.2. Polyphenols and Antioxidant Activities

Polyphenols in plants can act as antioxidants and protect cells from free radical damage, as their ability to scavenge free radicals helps maintain homeostasis [29]. The concentration of polyphenols in spray-dried tamarillo is summarised in Table 2. The results showed that chlorogenic acid and kaempferol rutinoside were the most abundant phenolic compounds in tamarillo powder (3.01 ± 0.51 to 7.11 ± 0.32 and 1.86 ± 0.11 to 4.03 ± 0.20 mg/100 g DW, respectively). This was consistent with results reported by Espin et al. [1], which reported chlorogenic acid as being the primary phenolic compound in tamarillo. The polyphenols are highly epimerase in response to a combination of high temperature and alkaline acid-base pH [18], which may account for the lower values obtained in the spray-dried powder.

**Figure A1:**
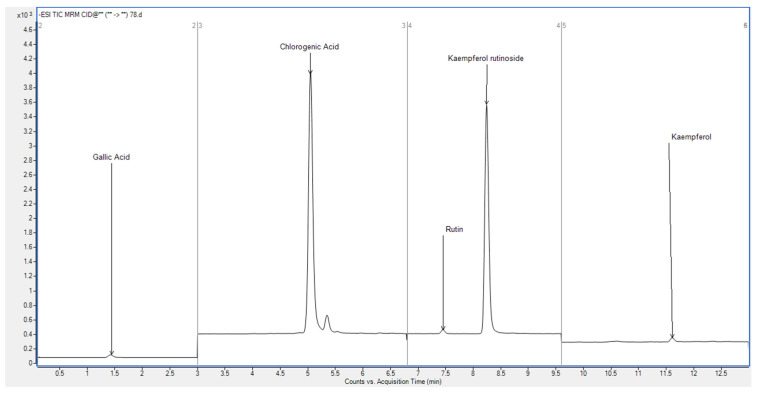
LC-MS profile of individual polyphenol compounds of spray-dried tamarillo powder. The run is divided to different segments for different ions.

**Figure A2:**
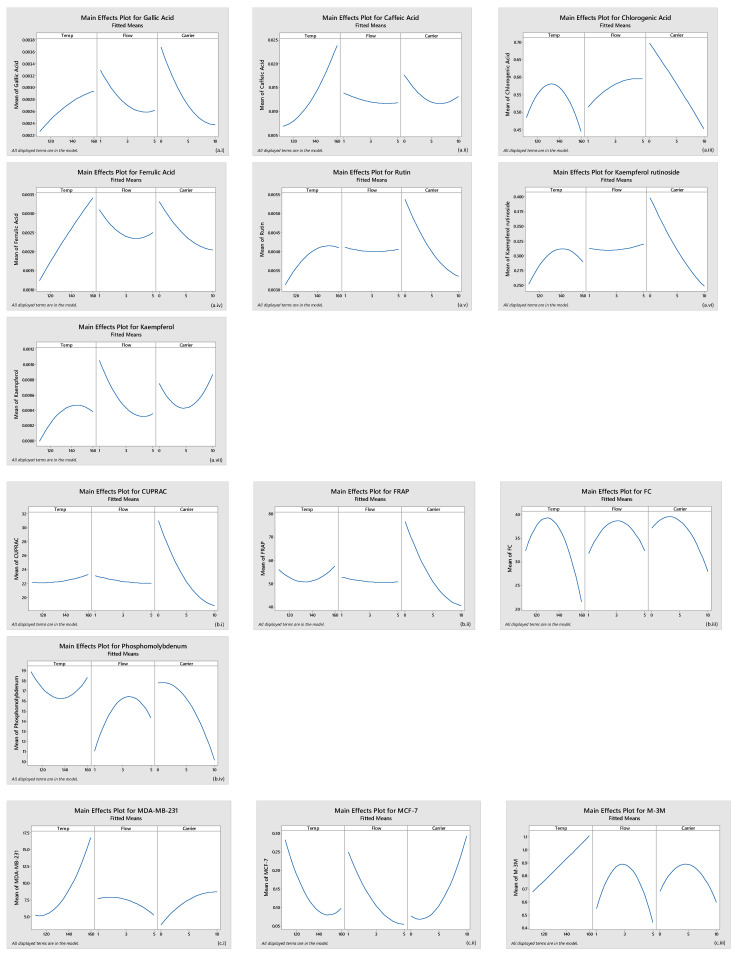
Main effect plots for total phenolic content, antioxidant activity, and anticancer activity.

The second-order models fit well with polyphenol content, where the three factors (air inlet temperature, flow rate, and carrier concentration) showed significant influence on the variation in the responses (Table 3). The LCMS chromatogram is shown in Figure A1 of Appendix A. The air inlet temperature, flow rate and carrier concentration had significant main effects on gallic acid and ferulic acid, followed by the quadratic effect of the flow rate and carrier for ferulic acid, and the interaction effect between the temperature and flow rate for gallic acid. With an increase in temperature, there was a significant increase in gallic acid, caffeic acid, ferulic acid, rutin, and kaempferol rutinoside (Figure A2). Mishra et al., [30] reported that spray-drying of *Emblica officinalis* juice powder with an air inlet temperature from 125 to 175 °C decreased the total phenolic content significantly (*p* < 0.001). However, when the temperature increased above 175 °C, the total phenolic content increased. The authors postulated that exposure to higher temperature resulted in polymerisation as well as synthesis of polyphenols compounds in their samples. In addition, the decomposition of polyphenols can result in some decomposition products that still have radical-scavenging activity [31]. The cyclo-inclusion effect of air inlet temperature, and the addition of maltodextrins, can influence the degradation and conversion of polyphenols [12].

**Figure 2:**
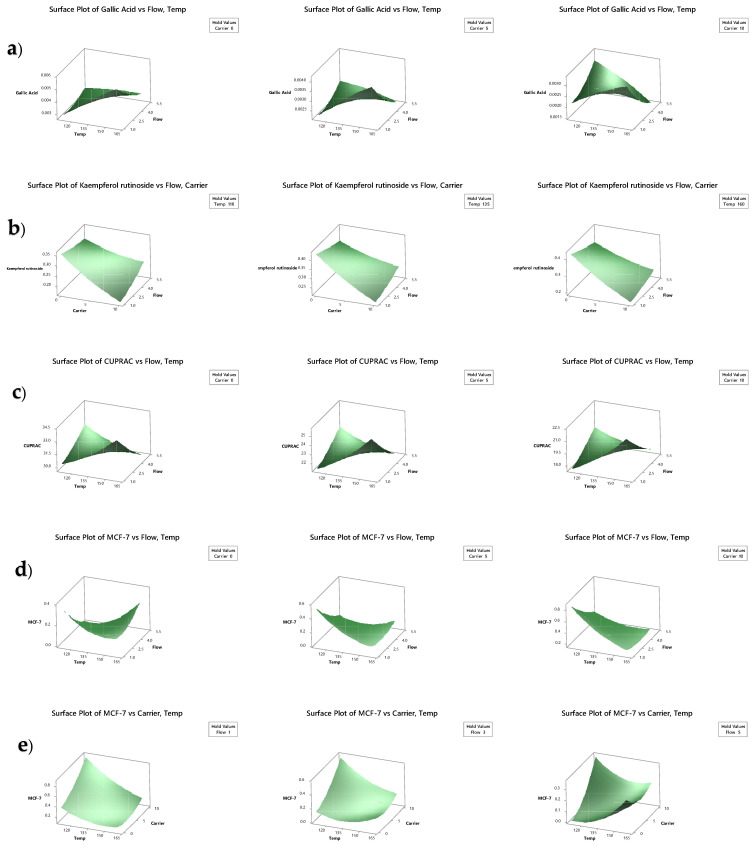
Response surface three-dimensional plots of significant interactions for gallic acid (**a**), kaempferol rutinoside (**b**), antioxidant capacity measured by the CUPRAC method (**c**), and IC50 values for MCF-7 cancer cell line (**d**,**e**) as a function of the studied factors.

An increase in flow rate significantly decreased gallic acid and ferulic acid (Figure A2). Georgetti et al. [32] found that the exposure of phenolics to the hot gas streams of spray-drying may result in oxidation reactions or heat-induced decomposition of thermolabile compounds, causing a degradation or loss of polyphenols and readily volatile substances. Gallic acid had a significant interaction effect with temperature and flow rate, as shown in Figure 2a. At a lower flow rate and a higher temperature, there was a higher concentration gallic acid at a 0% and 5% carrier concentration. However, at a 10% carrier concentration, the concentration of gallic acid was higher at both a low flow rate and a high temperature, as well as at a high flow rate and a low temperature. As seen in Table 2, caffeic acid, rutin, chlorogenic acid, and kaempferol rutinoside significantly (*p* < 0.05) increased with increased temperature. On the other hand, rutin, chlorogenic acid, and kaempferol rutinoside significantly (*p* < 0.05) decreased with increased carrier concentration. Moreover, kaempferol rutinoside showed a significant interaction effect with flow rate and carrier concentration. At a higher temperature, kaempferol rutinoside concentration was high with a low carrier concentration, which slowly decreased with increasing flow rate (Figure A2). Carrier materials can result in coating and dilution, which can reduce the polyphenol content [33].

### 3.3. Antioxidant Capacity, Total Polyphenolic, Total Flavonoid and Total Carotenoid Content

The high antioxidant capacity of tamarillo makes it a good source of natural dietary supplement [1,2]. The effect of spray-drying processing parameters on the antioxidant activities (CUPRAC, FRAP, and PM), as well as the total phenolic (F-C), total flavonoid (TF) and total carotenoid content (TCC) of tamarillo powder, were evaluated. The antioxidant capacity of the spray-dried powder was found to be within the range of 7.79 ± 0.99 to 91.05 ± 3.23 mg/g DW (Table 2) The second-order models fitted well to explain how antioxidant activities were influenced by the three spray-drying parameters, as shown in Table 3. Carrier concentration significantly influenced CUPRAC, FRAP, and PM antioxidant activities, followed by the quadratic effect of carrier concentration. With increasing carrier concentration, there was a significant decrease in all three antioxidant activities (Figure A2). Similarly, increasing the carrier concentration significantly decreased TF and TCC (Table 2 and Table 3). Maltodextrin reduced the total concentration of all phytochemicals in spray dried Moringa leaf juice powder by acting as a diluent [34].

In the present study, the spray-drying inlet temperature and carrier concentration significantly influenced total polyphenol content (F-C), followed by the quadratic effects of temperature, flow rate, and carrier concentration, as shown in Table 3. Increasing the temperature up to 140 °C increased total polyphenols. However, after 140 °C, the total polyphenol content (F-C) significantly increased (Figure A2 and Figure 1). It is postulated that heat treatment released bound soluble polyphenolic and flavonoid compounds [35]. In addition, a high concentration of carrier significantly reduced F-C. Similarly, the total polyphenolic content of amla (*Emblica officinalis*) powder was significantly reduced when the concentration of maltodextrin was increased from 5 to 9%, which was due to the concentration effect of maltodextrin [30].

### 3.4. Anticancer Capacity

The cytotoxic activities of tamarillo powder against MDA, MCF-7, and M-3M were evaluated using the MTT assay. The tamarillo extracts were tested between the concentration ranges of 0–5 mg/mL (Table 2). The results showed that tamarillo powder caused apoptosis in a dose-dependent manner. These results are similar to those reported by Mutalib et al. [3], where tamarillo extracts were found to have selective cytotoxicity to MDA-MB-231, and that the proliferation of this cancer cell line was in a dose-dependent manner. Mutalib et al. [3] found that the crude ethanol extract had the best IC50 at 80.00 ± 3.40 μg/mL for the MDA cell line. In the present study, the inhibitory effect of aqueous tamarillo extract was the lowest at 130.00 ± 3.60 μg/mL. The tamarillo powder had an inhibitory effect on cancer cells at high concentrations. *Rubus coreanus* Miquel (Korean blackberry) extract decreased neuronal PC-12 intracellular oxidative stress at low concentrations, increased cell viability, and resulted in inhibitory effects on cells only at high concentrations [36].

Table 3 presents the linear, quadratic, and interaction terms, which summarised the effect of spray-drying conditions on IC50 values. Inlet air temperature had the most significant effect on all three cancer cell lines (MDA-MB-231, M-3M, and MCF-7). With increasing temperature, there was a significant increase in IC50 values in the MDA-MB-231 and M-3M cell lines (Figure A2). However, for MCF-7, increasing temperature resulted in a significant decrease in IC50 values lines (Figure A2). Low IC50 values indicate that a low concentration is required to achieve 50% viability of cancer cells using the MTT assay. Hence, tamarillo powder produced at low temperatures had better anticancer effects for MDA-MB-231 and M-3M cell lines. As for MCF-7 cells, tamarillo powder produced at higher temperatures (140–160 °C) had a better inhibitory effect on cancer cells. Different pharmacological activities of the main plant components (phenols, flavonoids) in the tamarillo extracts may explain the cytotoxic activity on MCF-7 breast cancer cells [37]. Inlet temperature has been reported to influence the retention of phytochemicals such as plant total phenols and anthocyanins, resulting in different effects on cell proliferation inhibition [38].

### 3.5. Relationship between Anticancer Activity and Bioactive Compounds

**Figure 3:**
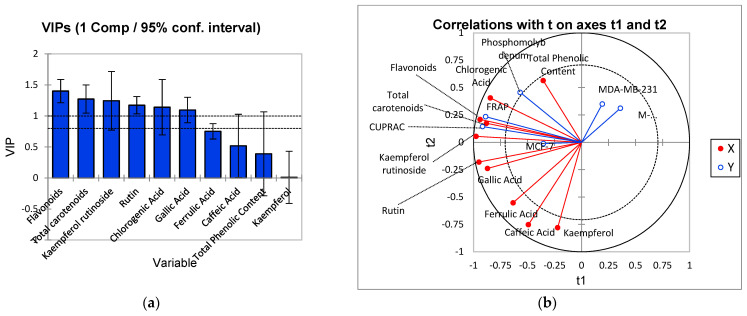
Partial Least Squares Regression (PLSR) analysis to determine the relationship between bioactive compounds and antioxidant activities with anticancer activities: (**a**) Variable Importance in Projection (VIP) plot; (**b**) PLSR correlation plot.

Variable Importance in Projection (VIP) values (Figure 3a) were used to determine the important polyphenolic compounds and antioxidant activities that contributed to the anticancer properties of tamarillo powder. A total of four polyphenols (chlorogenic acid, kaempferol rutinoside, gallic acid, and rutin) with VIP values > 1.0 were identified (Figure 2a). These polyphenols were the most important compounds that contributed to anticancer activities. Polyphenols can inhibit aberrant cell proliferation, which may lead to tumour cell apoptosis [1,12]. In addition, total flavonoid and total carotenoid content are also important contributors to the anticancer and antioxidant activities of spray-dried tamarillo powders (Figure 2b). The PLSR results (Figure 3b) further confirmed this relationship, where Dimension 1 and Dimension 2 explained a total variance of 45.84%. All polyphenols and antioxidant activities were negatively loaded along Dimension 1 and were correlated with the anticancer activity of the MCF-7 cell line. The different pharmacological activities of the main plant components (phenols, flavonoids) in plant extracts can contribute to cytotoxic activity in MCF-7 breast cancer cells [37]. Although tamarillo’s phenolic compound concentration is similar or lower than that of other fruits, it possesses greater antioxidant capacity than many commonly consumed antioxidant-rich fruits such as tomato [1]. Tree tomato fruits are rich in other antioxidants such as vitamin C and carotenoids [39], which can contribute to its antioxidant capacity. Hydroxycinnamoyl acids and rosmarinic acid, which are the major phenolic compounds in tree tomato, have been shown to possess higher scavenging activity than ascorbic acid and tocopherol [40].

### 3.6. Optimisation of Spray-Drying Parameters for Tamarillo Powders with Anticancer Activities

Optimisation was based on the lowest response value for IC50 and the highest response values for antioxidant, yield and bioactive compounds, to determine the best spray-drying conditions. The spray-drying conditions for drying tamarillo puree to produce a powder with high antioxidant and anticancer activity, a high yield, and stable bioactive compounds were optimised. The optimised conditions were at an inlet temperature of 146.8 °C and a flow rate of 1.76 g/mL, without carrier inclusion. However, the addition of a carrier may be necessary, as the SEM results showed that the inclusion of a carrier resulted in better particle morphology. Hence, in optimization, there should be a balance between maximising bioactivities and powder quality. Interestingly, carrier concentration in this study showed no significant effect on yield.

## 4. Conclusions

Spray-drying of tamarillo fruit puree using the response surface methodology (RSM) was carried out in this study. The effects of spray-drying conditions on the phenolic content, antioxidant activity, and anticancer properties of tamarillo fruit powder were determined. The anticancer activity of the MCF-7 cell line was correlated with polyphenols and antioxidant activities. For the MDA-MB-231 and MCF-7 cancer cell lines, spray-drying conditions had no significant effect on anticancer activities. However, for the M-3M cell line, anticancer activity was reduced with high temperature and the inclusion of a 10% carrier. Optimisation of the spray-drying parameters was carried out successfully on the different cancer cell lines, using RSM to maximise the bioactive compound content, antioxidant activity and anticancer activity. However, there needs to be a balance between maximising bioactivities and powder quality when it comes to optimising powder production. Further work can be carried out by exploring the use of conventional spray-drying in conjunction with the ultrasonic atomisation technique and dehumidifying air supply system, which may further improve tamarillo powder quality characteristics.

## Figures and Tables

**Table 1:** The tamarillo spray-drying experimental design used in this study summarising the different treatments according to the Box–Behnken design.

Experiment No.	Std Order	Run Order	Pt Type	Blocks	Temperature (°C)	Flow (g/mL)	Carrier (%)
1	1	1	2	1	110	1	5
2	13	2	0	1	135	3	5
3	7	3	2	1	110	3	10
4	2	4	2	1	160	1	5
5	12	5	2	1	135	5	10
6	10	6	2	1	135	5	0
7	9	7	2	1	135	1	0
8	14	8	0	1	135	3	5
9	5	9	2	1	110	3	0
10	3	10	2	1	110	5	5
11	8	11	2	1	160	3	10
12	11	12	2	1	135	1	10
13	4	13	2	1	160	5	5
14	15	14	0	1	135	3	5
15	6	15	2	1	160	3	0

**Table 2:** Profiles of polyphenol compounds (mg/100 g DW); antioxidant activity (CUPRAC: cupric reducing antioxidant capacity (mg Trolox/g DW), FRAP: ferric reducing antioxidant power (mg TE/g DW), PM: phosphomolybdenum total antioxidant capacity (mg TE/g DW)); total phenolics, flavonoids, and carotenoids (F-C: Folin–Ciocalteu reductive capacity (mg GAE/g DW), TF: total flavonoid content (mg QEs/g DW), TCC: the total carotenoid content (mg (β-carotene)/g)); and anticancer activity (MDA-MB-231: human breast cancer cell line, MCF-7: breast cancer cell line, M-3M: skin cancer cell line) of spray-dried tamarillo powder. Values are expressed as Mean ± SD (*n* = 6).

Condition	Total Phenolic Content							Antioxidant Activity			Total phenolics, Flavonoids, and Carotenoids			Anticancer Activity Activity		
	Gallic Acid	Caffeic Acid	Chlorogenic Acid	Ferulic Acid	Rutin	Kaempferol rutinoside	Kaempferol	CUPRAC	FRAP	PM	F-C	TF	TCC	MDA-MB-231	MCF-7	M-3M
110-1-5	0.023 ± 0.0034 ^de^	0.08 ± 0.022 ^gh^	4.58 ± 1.0 ^c^	0.018 ± 0.011 ^def^	0.032 ± 0.008 ^de^	2.62 ± 0.65 ^defg^	0.003 ± 0.0007 ^e^	21.52 ± 1.11 ^ef^	60.49 ± 3.23 ^d^	13.82 ± 0.94 ^bcde^	28.21 ± 1.44 ^bcd^	8.28 ± 0.46 ^c^	2.27 ± 0.06 ^ef^	2.79 ± 1.52 ^a^	0.56 ± 0.16 ^a^	0.42 ± 0.36 ^b^
110-3-10	0.024 ± 0.0014 ^de^	0.14 ± 0.0041 ^h^	3.19 ± 0.13 ^ef^	0.009 ± 0.0032 ^f^	0.025 ± 0.0017 ^e^	1.86 ± 0.11 ^h^	0.004 ± 0.0006 ^e^	17.48 ± 0.71 ^g^	43.98 ± 2.77 ^g^	12.32 ± 1.57 ^cde^	17.74 ± 2.19 ^efg^	6.56 ± 0.09 ^e^	2.09 ± 0.05 ^fg^	4.77 ± 1.79 ^a^	0.63 ± 0.05 ^a^	0 ± 0 ^b^
110-3-0	0.030 ± 0.0019 ^cd^	0.071 ± 0.010 ^ef^	6.59 ± 0.36 ^ab^	0.022 ± 0.011 ^cdef^	0.046 ± 0.0032 ^bc^	3.38 ± 0.13 ^bc^	0.004 ± 0.0004 ^e^	31.64 ± 1.03 ^ab^	80.37 ± 3.53 ^b^	20.76 ± 0.86 ^a^	33.24 ± 3.40 ^ab^	11.83 ± 0.29 ^b^	3.47 ± 0.06 ^b^	3.28 ± 0.35 ^a^	0.11 ± 0.01 ^a^	0.49 ± 0.23 ^b^
110-5-5	0.025 ± 0.0039 ^de^	0.078 ± 0.010 ^h^	4.39 ± 0.34 ^cd^	0.014 ± 0.070 ^ef^	0.031 ± 0.0053 ^de^	2.55 ± 0.23 ^defg^	0.003 ± 0.0007 ^e^	23.76 ± 0.62 ^cd^	56.90 ± 1.57 ^d^	17.08 ± 1.54 ^ab^	24.76 ± 3.58 ^cde^	8.52 ± 0.16 ^c^	2.77 ± 0.05 ^c^	2.64 ± 0.66 ^a^	0.1 ± 0.01 ^a^	0.17 ± 0.01 ^b^
135-3-5	0.027 ± 0.0035 ^de^	0.12 ± 0.021 ^fg^	5.84 ± 0.82 ^b^	0.024 ± 0.0061 ^bcdef^	0.040 ± 0.0067 ^cd^	3.06 ± 0.49 ^cde^	0.004 ± 0.0015 ^de^	22.28 ± 0.99 ^de^	50.74 ± 1.43 ^ef^	16.27 ± 0.92 ^bc^	38.72 ± 2.35 ^a^	8.78 ± 0.47 ^c^	2.78 ± 0.14 ^c^	2.05 ± 0.27 ^a^	0.49 ± 0.2 ^a^	0.4 ± 0.15 ^b^
135-5-10	0.040 ± 0.035 ^e^	0.22 ± 0.039 ^bc^	3.48 ± 0.53 ^def^	0.028 ± 0.0059 ^abcdef^	0.035 ± 0.0054 ^de^	2.44 ± 0.37 ^efgh^	0.004 ± 0.0012 ^de^	19.38 ± 0.40 ^fg^	45.80 ± 1.42 ^fg^	10.02 ± 1.20 ^ef^	26.20 ± 3.21 ^bcd^	7.36 ± 0.38 ^d^	2.33 ± 0.09 ^de^	6.83 ± 2.04 ^a^	0.11 ± 0.01 ^a^	0.16 ± 0.14 ^b^
135-5-0	0.036 ± 0.0022 ^bc^	0.17 ± 0.0075 ^de^	7.11 ± 0.32 ^a^	0.031 ± 0.0091 ^abcde^	0.052 ± 0.0054 ^ab^	3.83 ± 0.22 ^ab^	0.007 ± 0.0017 ^cd^	29.75 ± 0.85 ^b^	72.72 ± 2.39 ^c^	15.62 ± 0.70 ^bc^	27.31 ± 1.34 ^bcd^	11.22 ± 0.20 ^b^	3.42 ± 0.16 ^b^	5.52 ± 1.31 ^a^	0.16 ± 0.02 ^a^	0.29 ± 0.05 ^b^
135-1-0	0.041 ± 0.0009 ^ab^	0.13 ± 0.0074 ^ef^	5.77 ± 0.37 ^b^	0.041 ± 0.016 ^abc^	0.053 ± 0.0041 ^ab^	4.10 ± 0.20 ^a^	0.008 ± 0.0008 ^c^	31.12 ± 1.28 ^ab^	76.39 ± 1.61 ^bc^	10.92 ± 0.55 ^def^	29.20 ± 2.92 ^bc^	11.44 ± 0.19 ^b^	3.38 ± 0.06 ^b^	3.78 ± 0.12 ^a^	0.18 ± 0.07 ^a^	0.19 ± 0.02 ^b^
135-1-10	0.027 ± 0.0032 ^de^	0.23 ± 0.033 ^bc^	3.28 ± 0.43 ^ef^	0.027 ± 0.0068 ^abcdef^	0.033 ± 0.0051 ^de^	2.24 ± 0.33 ^fgh^	0.024 ± 0.0041 ^a^	20.42 ± 0.32 ^ef^	43.47 ± 4.39 ^g^	7.79 ± 0.99 ^f^	21.06 ± 1.77 ^def^	6.79 ± 0.27 ^de^	2.32 ± 0.11 ^def^	6.82 ± 0.11 ^a^	0.25 ± 0.06 ^a^	0.26 ± 0.07 ^b^
160-1-5	0.045 ± 0.011 ^a^	0.25 ± 0.028 ^b^	4.51 ± 0.55 ^c^	0.042 ± 0.012 ^ab^	0.046 ± 0.006 ^bc^	3.11 ± 0.38 ^cd^	0.010 ± 0.0013 ^bc^	25.37 ± 1.89 ^c^	58.35 ± 2.47 ^d^	14.60 ± 0.50 ^bcd^	12.91 ± 0.42 ^gh^	8.84 ± 0.26 ^c^	1.95 ± 0.19 ^g^	7.67 ± 6.87 ^a^	0.48 ± 0.6 ^a^	0.55 ± 0.48 ^b^
160-3-10	0.020 ± 0.0023 ^e^	0.19 ± 0.031 ^cd^	3.01 ± 0.51 ^f^	0.028 ± 0.009 ^abedef^	0.030 ± 0.006 ^de^	2.06 ± 0.39 ^gh^	0.003 ± 0.0009 ^e^	19.44 ± 0.64 ^fg^	43.14 ± 2.04 ^g^	10.84 ± 1.38 ^def^	9.96 ± 0.64 ^h^	6.50 ± 0.35 ^e^	1.98 ± 0.17 ^g^	2.19 ± 0.46 ^a^	0.2 ± 0.03 ^a^	32.47 ± 55.64 a^b^
160-5-5	0.021 ± 0.0006 ^e^	0.24 ± 0.013 ^b^	4.10 ± 0.28 ^cde^	0.035 ± 0.014 ^abcd^	0.039 ± 0.0033 ^cd^	2.82 ± 0.17 ^cdef^	0.003 ± 0.0005 ^e^	21.99 ± 2.13 ^ef^	55.37 ± 3.82 ^de^	14.76 ± 1.94 ^bcd^	15.40 ± 1.62 ^fgh^	8.98 ± 0.75 ^c^	2.54 ± 0.10 ^cd^	1.42 ± 1.29 ^a^	0.19 ± 0.06 ^a^	0.73 ± 0.17 ^b^
160-3-0	0.043 ± 0.0044 ^ab^	0.36 ± 0.019 ^a^	5.62 ± 0.30 ^b^	0.045 ± 0.011 ^a^	0.058 ± 0.005 ^a^	4.03 ± 0.20 ^a^	0.012 ± 0.0008 ^b^	32.72 ± 1.09 ^a^	91.05 ± 3.23 ^a^	21.44 ± 1.01 ^a^	22.15 ± 4.16 ^cdef^	13.23 ± 0.32 ^a^	4.71 ± 0.20 ^a^	4.86 ± 2.37 ^a^	0.16 ± 0.05 ^a^	149.79 ± 143.15 ^a^

^a–h^ Data are shown as statistical significance of tamarillo phenolics (*p* < 0.05). CUPRAC: cupric reducing antioxidant capacity (mg Trolox/g DW), FRAP antioxidant activity: ferric reducing antioxidant power (mg TE/ g DW), F-C: Folin–Ciocalteu reductive capacity (mg GAE/g DW), PM: phosphomolybdenum total antioxidant capacity (mg TE/g DW), TF: total flavonoid content (mg QEs/g DW), TCC: the total carotenoid content (mg (β-carotene)/g) across different processing conditions.

**Table 3:** The two-way ANOVA summarising the regression models and regression coefficients obtained for antioxidant activity (CUPRAC: cupric reducing antioxidant capacity (mg Trolox/g DW), FRAP: ferric reducing antioxidant power (mg TE/g DW), PM: phosphomolybdenum total antioxidant capacity (mg TE/g DW)); total phenolics, flavonoids, and carotenoids (F-C: Folin–Ciocalteu reductive capacity (mg GAE/g DW), TF: total flavonoid content (mg QEs/g DW), TCC: the total carotenoid content (mg (β-carotene)/g)); and anticancer activity (MDA-MB-231: human breast cancer cell line, MCF-7: breast cancer cell line, M-3M: skin cancer cell line) of spray-dried tamarillo powder.

Regression Coefficients	Total Phenolic Content							Antioxidant Activity			Total Phenolics, Flavonoids, and Carotenoids			Anticancer Activity		
	Gallic Acid	Caffeic Acid	Chlorogenic Acid	Ferulic Acid	Rutin	Kaempferol rutinoside	Kaempferol	CUPRAC	FRAP	PM	F−C	TF	TCC	MDA−MB−231	MCF−7	M−3M
**Intercept**																
**X1_0_**	−0.00737	0.0516	−2.55	−0.0042	−0.01018	−0.894	−0.00952	30.4	240.2	69.0	−273.3	10.83	−1.86	70.0	3.66	−1.12
**Linear**																
**X_1_**	0.000113 *	−0.001 **	0.0484	0.000083 ***	0.000203 *	0.01834 *	0.000132	−0.077	−2.47	−0.969	4.751 **	−0.016	0.058	−1.213 *	−0.0433 *	0.0065 *
**X_2_**	0.001149 *	0.00055	0.056	−0.000629 **	0.000161	−0.0022	0.000108	3.54	−3.15	8.24 *	5.18	0.354	0.462	7.15	−0.425 **	0.267
**X_3_**	0.000161 **	0.00231	−0.0531 **	−0.000175 ***	−0.000218 ***	−0.0187 ***	0.000235	−2.52 ***	−4.09 ***	0.716 ***	0.12 **	−0.437 ***	0.058 **	−2.88	0.1123 **	0.261
**Square**																
**X_1_^2^**	0	0.000005	−0.000184 *	0	−0.000001	−0.000062 *	0	0.000686	0.00966 *	0.00380 *	−0.01886 ***	0.000162	−0.000158	0.00551	0.000135	0.000004
**X_2_^2^**	0.000063	0.000212	−0.0062	0.000099 **	0.000022	0.00156	0.000068	0.069	0.250	−0.894 **	−1.654 **	−0.0566	−0.0747	−0.276	0.01168	−0.0981 **
**X_3_^2^**	0.000013	0.000136	−0.00020	0.000011 *	0.000014	0.00055	0.000015	0.1045 **	0.3142 *	−0.0921 *	−0.2465 *	0.0260	0.01525	−0.0523	0.00319	−0.00991 *
**Two−way interaction**																
**X_12_**	−0.000013 *	−.000003	−0.0001	−0.000001	−0.000003	−0.00015	−0.000003	−0.0316 *	0.0031	−0.0155	0.0297	−0.00050	0.00045	−0.0537	0.002408 *	0.00232
**X_13_**	−0.000003	−0.000022	0.00016	−0.000001	−0.000001	−0.00008	−0.000002	0.00176	−0.0230	−0.00432	0.0066	−0.00292	−0.00270	0.0237	−0.000825 *	−0.001185
**X_23_**	0.000012	−0.000375	0.003	0.000018	0.00002	0.003 *	−0.000047	0.0083	0.150	0.0080	0.176	0.0197	−0.0007	0.230	−0.00373	−0.00359
**R^2^**	0.94	0.88	0.86	0.99	0.95	0.97	0.82	0.98	0.96	0.95	0.96	0.97	0.87	0.84	0.95	0.71
**F value (model)**	9.12 *	4.33	3.42	70.57 ***	10.14 **	17.98 **	2.46	34.05 **	16.44 ***	10.76 **	13.10 **	20.22 **	3.75	2.86	10.55 **	4.76

X_1_: temperature (°C), X_2_: flow rate (g/mL), X_3_: carrier concentration (%). Level of significance: * *p* < 0.05, ** *p* < 0.01, *** *p* < 0.001. R^2^: coefficient of determination.

## Data Availability

Data is not available upon request.

## Sample Availability

Samples of the compounds are not available from the authors.
